# Estimating the Prevalence of Opioid use Disorder in the Cincinnati Region using Probabilistic Multiplier Methods and Model Averaging

**DOI:** 10.36469/9729

**Published:** 2019-04-03

**Authors:** Peter J. Mallow, Nila Sathe, Michael Topmiller, Jennifer Chubinski, Dillon Carr, Roni Christopher

**Affiliations:** 1 Xavier University, Cincinnati, OH; 2 Premier, Inc., Charlotte, NC; 3 Premier, Inc., Charlotte, NC and 4American Academy of Family Physicians, Cincinnati, OH; 4 Interact for Health, Cincinnati, OH

**Keywords:** addiction, substance use disorder, multiplier method, simulation model, prevalence, cincinnati region, opioid use disorder

## Abstract

**Background:** Opioid use disorder (OUD) and its consequences have strained the resources of health, social, and criminal justice services in the Cincinnati region. However, understanding of the potential number of people suffering from OUD is limited. Little robust and reliable information quantifies the prevalence and there is often great variation between individual estimates of prevalence. In other fields such as meteorology, finance, sports, and politics, model averaging is commonly employed to improve estimates and forecasts. The objective of this study was to apply a model averaging approach to estimate the number of individuals with OUD in the Cincinnati region.

**Methods:** Three individual probabilistic simulation models were developed to estimate the number of OUD individuals in the Cincinnati Core Based Statistical Area (CBSA). The models used counts of overdose deaths, non-fatal overdoses, and treatment admissions as benchmark data. A systematic literature review was performed to obtain the multiplier data for each model. The three models were averaged to generate single estimate and confidence band of the prevalence of OUD.

**Results:** This study estimated 15 067 (SE 1556) individuals with OUD in the Cincinnati CBSA (2 165 139 total population). Based on these results, we estimate the prevalence of OUD to be between 13 507 (0.62% of population) and 16 620 (0.77% of population).

**Conclusions:** The method proposed herein has been shown in diverse fields to mitigate some of the uncertainty associated with reliance on a single model. Further, the simplicity of the method described is easily replicable by community health centers, first-responders, and social services to estimate capacity needs supported by OUD estimates for the region they serve.

## Background

Opioid use in the United States (US) has reached staggering proportions. The 2016 National Survey on Drug Use and Health conducted by the Substance Abuse and Mental Health Services Administration (SAMHSA) reported that 11.8 million individuals ages 12 or older, 4.4% of the population in this age range, misused opioids.^1^ Recent opioid prescribing guidelines have recommended restrictions and stepped approaches to prescribing,^2,3^ but even as the number of opioid prescriptions has declined,^4,5^ the number of opioid-related overdoses remains high. Predicted estimates for drug overdose deaths in 2017 exceed 70 000, with opioid-associated deaths accounting for the majority.^6^

Reasons for the epidemic of misuse are multifaceted and include a focus on pain management as a key aspect of patient care and attempts of pharmaceutical companies to promote opioids as minimally addictive, leading to subsequent overprescribing.^7,8^ Some areas of the country, in particular parts of Ohio, Kentucky, and southeast Indiana, have been particularly affected by OUD. The number of overdose deaths per 10 000 in population reported in 2017 were: 2.7; 3.4; and 4.2 for Indiana, Kentucky, and Ohio, respectively, whereas the overdose death rate for United States was 1.9.^6^ In Hamilton County, the largest county in the Cincinnati Core Based Statistical Area (CBSA), the overdose deaths per 10 000 was 6.5 in 2017.^9^

Understanding the prevalence of OUD in a given area is key for planning approaches and services to address the problem. Such understanding, however, is complicated by several factors. Data on opioid-related issues (prescribing, overdoses, deaths) are often seemingly contradictory given variations in data sources.^10^ National surveys addressing opioid use often use variable terminology and methods, which may create conflicting estimates of prevalence when trying to compare across studies.^11^ Moreover, survey data is subject to well-known limitations of sampling and reporting bias.^12^ Granular data, such as at the state or county level sources may be non-existent,^13^ though some states and cities have developed their own dashboards to track different issues. The City of Cincinnati, for example, makes opioid overdosing tracking publicly accessible.^14^ However, comprehensive estimates are often based on a single model using best-available data, such as overdoses or treatment admissions. Differing models may create contradictory estimates or such broad ranges potentially limiting their applicability to first-responders, social services, and criminal justice services trying to understand demand for their services.^15^

Model averaging has been shown to be a more robust approach, though rarely applied to estimate the prevalence of substance abuse disorders, including OUD.^15–18^ Model averaging approaches have included combining the results of different models each with their own structural or data instability and have been shown to be more beneficial than relying on a single model.^19–21^ The method described in this study allowed for an estimate of the total number of individuals in the Cincinnati region who may be suffering from OUD while controlling for model and parameter uncertainty. Generating fundamental knowledge about the size of the OUD population, in the face of limited resources, will enable the criminal justice, social service, and healthcare systems to assess the total need for services required to address the opioid epidemic.

## Methods

### Overview of Model Development

We developed three probabilistic multiplier models to estimate the prevalence of OUD in the Cincinnati region for 2017. The models were then averaged together to generate a final estimate of the prevalence. The three individual models were: 1. overdose deaths, 2. treatment admissions, and 3. non-fatal emergency department (ED) opioid visits (data sources described below). The resulting estimates of OUD prevalence were averaged to estimate the number of individuals with OUD in the Cincinnati region. The Cincinnati region was defined by the US Census Bureau’s definition of the Cincinnati CBSA.^22^ The Cincinnati CBSA is comprised of 14 counties in Indiana (Dearborn, Franklin, Ohio), Kentucky (Boone, Bracken, Campbell, Gallatin, Grant, Kenton, Pendleton), and Ohio (Clermont, Butler, Hamilton, Warren).

### Individual Multiplier Model Data

The three individual multiplier models used counts of the primary endpoint collected by different sources for the Cincinnati CBSA. Overdose deaths were obtained from the CDC WONDER Mortality Database (CDC Wonder).^23^ The CDC WONDER database contains detailed cause of death information reported by International Classification of Diseases, 10^th^ revision (ICD-10) codes. The following ICD-10 codes were used to identify fatal opioid related deaths for the year 2017: X40-44, X60-64, X85, Y10-14, and T40.0-0.4.

Treatment admission data was obtained from the Substance Abuse and Mental Health Services Administration Treatment Episode Data Set: Admissions (TEDS-A) for 2016.^24^ The TEDS-A database contains admissions to substance abuse treatment facilities for opioid-related causes. No adjustment was made for repeat treatment admissions within the same year. Most treatment programs last six to twelve months and, combined with wait times to enter treatment often exceeding several months, we assumed an individual will not enter treatment more than once in a year.^13^

The third model, non-fatal emergency department (ED) opioid visits for the year 2017, were obtained from several local sources and summed together to generate a gross number for the Cincinnati CBSA. Indiana counties were obtained from the state department of health.^25^ The Saint Elizabeth Healthcare system, Northern Kentucky’s (NKY) primary health system, provided counts for the NKY counties.^26^ In Ohio, county level non-fatal ED visits were obtained from Livestories Overdose Reporting Tool for Hamilton, Clermont, and Butler counties.^27^ Data were not available for Warren County Ohio. The total number of ED Visits was reduced by 35% to account for multiple ED visits by the same person.^28,29^

### Multiplier Data for the Individual Models

A “best evidence” literature review was conducted to identify multiplier data for each of the three models.^30^ The review consisted of a search of English language, US conducted peer-reviewed literature from January 2013 to August 2018 and indexed in MEDLINE (searched via PubMed). Included studies had to address the specific endpoint of the model and provide an estimate of the multiplier. For example, for the death model, only studies that included an estimate of the annual likelihood of dying from an opioid overdose were included in the results. The relevance of articles was assessed based on sample size, recentness of the article, and number of citations of the study. A patient weighted pooled analysis was conducted to obtain the average and standard error for each of the three endpoints.^30^

### Multiplier Modeling

Multiplier methods to estimate prevalence are among the most commonly used approaches.^31^ The multiplier method uses a known relevant endpoint A, an estimate of the proportion resulting in the endpoint B and the estimated total C. Knowledge of any two of the points allows the third to be calculated. For example, if the endpoint A were 1000 overdose deaths, and it was estimated that 5% of those with OUD will die annually (B), we estimate 20 000 people have OUD (C). Alternatively, if the population *C* and the multiplier *B* is known, one can estimate an unknown endpoint *A*.

The formula takes the following forms:

*C* = *A*/*B* or C*B = *A*

Where *A* equals the known endpoint, *B* equals the multiplier, and *C* equals the total population.

In this study, an expanded multiplier method was used by introducing a probabilistic modeling approach and model averaging of three individual models; typically, two of the three are assumed to be known.^19,31^ However, this is not often the case as the multiplier data may be based on a small sample, survey, or other estimate not necessarily generalizable to the region being examined. Multiplier B is often uncertain because it is frequently obtained from surveys or sample data. A Monte-Carlo simulation was employed to account for this uncertainty. The Monte-Carlo simulation assumed a beta distribution of the multiplier data using the average and standard error and the simulation was performed 10 000 times for each model.

### Model Averaging

The three individual models were averaged to estimate the total number of individuals with OUD in the Cincinnati region. A Monte-Carlo simulation addressed 1st order (parameter) and 2nd order (structural) uncertainty. The simulation was performed 10,000 times, and the average and standard error were reported. All calculations were performed with Microsoft Excel (Redmond, WA) and verified using Treeage Software (Williamstown, MA) to ensure computations yielded the same results. The Xavier University Institutional Review Board waived this research from review.

## Results

The individual multiplier model endpoints for the Cincinnati CBSA are shown in Table 1. Across the 14 counties of the Cincinnati CBSA, the population was 2 165 139. Reported opioid-related overdose deaths totaled 996, and 2752 individuals were admitted for opioid-related substance abuse treatment. Finally, 7342 non-fatal ED overdose visits occurred in the study period.

**Table 1.** Individual Multiplier Model Endpoint Data for Cincinnati MSA

**Parameter Name**	**Value**
Fatal Overdose^a^	996
Treatment Admission^b^	2752
Non-Fatal ED Overdose^c,d,e^	7342

^a^ CDC Wonder, 2018; ^b^ Substance Abuse and Mental Health Services Administration, 2018; ^c^ Indiana State Department of Health, 2018; ^d^ St. Elizabeth Health Care, 2018; ^e^ LiveStories.com

The results of the systematic literature review and patient weighted pooled analysis are shown in Table 2. A total of 10 studies including 318 504 individuals met the inclusion criteria and quality review. For the fatal overdose model, the patient weighted average value for the multiplier was 7.182% (SE 0.533%). The treatment admission pooled analysis found a patient weighted average of 21.376% (SE 2.142%). The patient weighted pooled results for the non-fatal ED overdose model was 40.89% (SE 5.111%).

**Table 2.** Multiplier Data for Use in Individual Models

**Parameter Name**	**Number** **of Studies**	**Number of Individuals**	**Multiplier Value, %**	**Std. Error, %**	**References**
Fatal Overdose	3	76 635	7.182	0.533	19,20,21
Treatment Admission	3	76 084	21.376	2.142	18,19,20
Non-Fatal ED Overdose	4	4243	40.890	5.111	19,20,28,29

The multiplier value and standard error (Std. Error) calculated through a patient weighted pooled analysis.

The individual model results found the population of those with OUD in the Cincinnati CBSA ranges from 9607 to 27 477 (Table 3). These results reflect 10 000 simulation iterations, varying the multiplier parameter based on a beta distribution created from the corresponding multiplier average and standard error. The fatal overdose model had the smallest spread between the minimum estimate, 11 342, and maximum estimate, 17 089. The average result of the 10 000 iterations was 13 944 (SE 996). The non-fatal ED overdose model had the highest spread between the minimum estimate, 13 206, and maximum estimate, 27 477. Of note, all three models had significant overlap. The median estimates ranged from 12 795 (treatment admission) to 17 972 (non-fatal ED overdose), while the mean estimates ranged from 12 968 (treatment admission) to 18 290 (non-fatal ED overdose).

**Table 3.** Multiplier Model Results

**Model**	**Min.**	**10th** **Percentile**	**50th** **Percentile**	**90th** **Percentile**	**Max.**	**Mean**	**SE**
Fatal Overdose	11 342	12 662	13 897	15 230	17 089	13 944	996
Treatment Admissions	9607	11 394	12 821	14 574	18 530	12 968	1284
Non-Fatal ED Overdoses	13 206	15 531	17 972	21 571	27 477	18 290	2390
**Model Average Results**	**11 382**	**13 189**	**14 888**	**17 121**	**21 029**	**15 067**	**1556**

ED: emergency department; SE: Standard Error The individual models reflect the result of a Monte-Carlo simulation of 10 000 iterations. The Model Average results are the average result of the three individual models equally weighted and a Monte-Carlo simulation of 10 000 iterations. All results are rounded to up to the nearest whole number

The average of the three models resulted in an estimate of 15 067 (SE 1556) individuals with OUD in the Cincinnati CBSA. Based on these results, we estimate the prevalence of OUD to be between 13 507 (0.62% of population) and 16 620 (0.77% of population). All results were replicated using TreeAge Software.

## Discussion

Estimating the number of individuals with OUD is an inherently difficult problem. The standard of directly surveying the OUD population is fraught with challenges including cost of surveys and identifying people with OUD. Those with OUD are less likely to respond to surveys and are not likely to be in contact with any social or criminal service unless necessary.^32^ The use of a single multiplier model may produce an inaccurate result or one with such wide variation that it has no utility for policy makers or the public service systems. Model averaging has an extensive literature in diverse fields ranging from finance, agriculture, politics, sports, and meteorology.^33–36^ The findings from these fields indicate that the use of model averaging reduces dispersions of the estimates and generally performs better than relying on a single model. The probabilistic model averaging approach to estimate OUD prevalence described herein is an easy-to-adopt method. It has been shown in other fields to mitigate some of the uncertainty of individual models and may improve the reliability of OUD prevalence estimates. Thus, it may prove to be a cost-effective method to improve estimates of OUD prevalence. Our findings indicate that there were 15 067 (SE 1556) individuals with OUD in the 14 county region in 2017. To the authors’ knowledge, this is the first comprehensive estimate of OUD prevalence for the Cincinnati CBSA.

The social, criminal justice, and healthcare resources in the region require reliable estimates of the OUD to allocate resources effectively and benchmark their activities. Yet, their resources for estimating their OUD population are constrained due to the necessity of funneling all resources to carry out their missions. Other indirect methods, such as capture-recapture or principal component regression analysis (PCR), are time consuming and require sophisticated analytical software and considerable financial resources to carry out. The method described herein is easily replicable.

Current, comprehensive estimates of the OUD population are non-existent, which fosters a continual budget strain on the government and funding agencies, who must respond to the individual problems of OUD in the absence of an understanding of the true scope of the problems. These problems include emergency medical service (EMS) calls for overdoses, naloxone distribution, treatment beds available, or forming quick response teams (QRTs). Local government and funding agencies can easily update OUD estimate results as new endpoint data and multiplier data become available. The method and results can be applied by community health centers, first-responders, and social services, to estimate capacity needs supported by OUD estimates for the region they serve.

### Limitations

The findings of this study must be considered in the context of the limitations of the methods. The first limitation is the findings are based on multiplier estimates from the peer-reviewed literature. The multiplier estimates were obtained from a systematic literature review and pooled to generate an average and standard error. The generalizability of these estimates to the Cincinnati region is assumed, and limitations mitigated in part by using a probabilistic modeling methodology and averaging the four models. The endpoint data for each of the four models relied on reported counts for a single year. Further, these counts, while accurate, may represent an under- or over- count due to reporting errors based upon the data of the underlying endpoint data.

Despite these limitations, few prevalence estimates of OUD are based on strong evidence, and policy makers and first responders must request resources and funding based on these inaccurate methods. The approach described in this study illustrated an approach to mitigating two significant sources of error: reliance on a single model and a parameter uncertainty. By addressing these two sources of error, we provide a more robust estimate of the prevalence of OUD in the Cincinnati CBSA.

## Conclusion

The idea of averaging multiple models is common in other fields, yet rarely applied when estimating the prevalence of substance abuse populations. The approach described in this study provides an easy and cost-effective method to generate estimates of OUD prevalence. The findings for the Cincinnati region may enable health, social, and criminal justice services to better address the opioid epidemic. Though costly, future research should attempt to validate these findings with direct methods of estimation.

## Abbreviations

**Table:** Abbreviations

CBSA	Core Based Statistical Area
CDC	Center for Disease Control and Prevention
ED	Emergency Department
EMS	Emergency Medical Services
ICD-10	International Classification of Diseases, 10th Revision
NKY	Northern Kentucky
PCR	Principal Component Regression Analysis
QRT	Quick Response Team
OUD	Opioid Use Disorder
SAMHSA	Substance Abuse and Mental Health Services Administration
SE	Standard Error
TEDS-A	Treatment Episode Data Set: Admissions
US	United States

## Availability of data and material

All data generated or analyzed during this study are included in this published article.

## Funding

Financial support for this research was provided in part by the Hamilton County, Ohio prosecutor’s office. The prosecutor’s office had no input on the study design, analysis, results, or the manuscript.

## Competing Interests

The authors declare that they have no competing interests to report with respect to this research.

## Authors’ contributions

Peter J. Mallow conceptualized the study design, conducted the analysis and systematic literature review, interpreted the results, and prepared the first draft of the manuscript. Michael Topmiller, Nila Sathe, Jennifer Chubinski, and Roni Christopher conceptualized the study design and interpreted the results. Dillon Carr and Peter J. Mallow conducted the systematic literature review. All authors have contributed substantially to the final version of the manuscript and have approved it in its final form.

## Acknowledgments

The authors wish to thank Kristin Dale for her thoughtful comments and edits during the development of this manuscript.
